# Factors in Initial Anticoagulation Choice in Hospitalized Patients With Pulmonary Embolism

**DOI:** 10.1001/jamanetworkopen.2024.52877

**Published:** 2025-01-03

**Authors:** William B. Stubblefield, Ron Helderman, Natalie Strokes, Colin F. Greineder, Geoffrey D. Barnes, David R. Vinson, Lauren M. Westafer

**Affiliations:** 1 Department of Emergency Medicine, Vanderbilt University Medical Center, Nashville, Tennessee; 2 Department of Emergency Medicine, The University of Texas Southwestern Medical Center, Dallas; 3 Department of Emergency Medicine, University of Massachusetts Chan Medical School-Baystate, Springfield; 4 Department of Emergency Medicine, University of Michigan, Ann Arbor; 5 Frankel Cardiovascular Center, Department of Internal Medicine, University of Michigan Medical School, Ann Arbor; 6 Division of Research, Kaiser Permanente Northern California, Pleasanton; 7 Department of Emergency Medicine, Kaiser Permanente Roseville Medical Center, Roseville, California; 8 Department of Healthcare Delivery and Population Science, University of Massachusetts Chan Medical School-Baystate, Springfield

## Abstract

**Importance:**

Despite guideline recommendations to use low-molecular-weight heparins (LMWHs) or direct oral anticoagulants in the treatment of most patients with acute pulmonary embolism (PE), US-based studies have found increasing use of unfractionated heparin (UFH) in hospitalized patients.

**Objective:**

To identify barriers and facilitators of guideline-concordant anticoagulation in patients hospitalized with acute PE.

**Design, Setting, and Participants:**

This qualitative study conducted semistructured interviews from February 1 to June 3, 2024, that were recorded, transcribed, and analyzed in an iterative process using reflexive thematic analysis. Interview participants were physicians in emergency medicine, hospital medicine (hospitalist), interventional cardiology, and interventional radiology. Participants were recruited using maximum variation sampling targeting UFH-dominant vs LMWH-dominant approaches in hospitalized patients with acute PE. We triangulated results with a group of interventional cardiologists and radiologists (interventionalists).

**Main Outcomes and Measures:**

Common themes and factors associated with anticoagulant selection for hospitalized patients with acute PE. Reflexive thematic analysis was used to identify these themes and factors.

**Results:**

Of the 46 interviewees (median [IQR] age, 43 [36-50] years; 33 who identified as men [71.7%]), 25 (54.3%) were emergency physicians, 17 (37.0%) were hospitalists, and 4 (8.7%) were interventionalists. Each interview lasted a median (IQR) of 29 (25-32) minutes. Prominent themes associated with anticoagulant selection included agnosticism regarding choice of anticoagulant, the inertia of learned practice, and therapeutic momentum after anticoagulation initiation. Institutional culture and support were factors associated with choice of the dominant anticoagulation strategy. Additionally, factors associated with UFH use were fear of decompensation and misperceptions regarding the pharmacology of anticoagulants and catheter-directed treatments.

**Conclusions and Relevance:**

In this qualitative study, physicians across a spectrum of specialties and geographical settings reported common barriers and facilitators to the use of guideline-concordant anticoagulation in patients hospitalized with acute PE, particularly agnosticism regarding choice of anticoagulant, inertia of learned practice, therapeutic momentum after anticoagulation initiation, and institutional culture and support. Future implementation efforts may consider targeting these domains.

## Key Points

**Question:**

What are factors associated with initial anticoagulation choice in hospitalized patients with acute pulmonary embolism (PE)?

**Findings:**

In this qualitative study of 46 physicians, major barriers and facilitators of using unfractionated heparin or low-molecular-weight heparin included agnosticism to choice of anticoagulant, inertia of learned practice, therapeutic momentum after anticoagulation was initiated, and institutional culture and support.

**Meaning:**

Findings of this study identify potential targets for implementation strategies for guideline-concordant anticoagulation in patients with acute PE.

## Teaser

This qualitative study assesses factors associated with the selection of unfractionated heparin vs low-molecular-weight heparin by emergency physicians, hospitalists, and interventional cardiologists and radiologists participating in a semistructured interview.

## Introduction

The annual incidence of pulmonary embolism (PE) in North America and Europe is approximately 60 to 120 per 100 000 persons.^1,2,3^ Patients are most commonly diagnosed with PE in the emergency department (ED), and the majority of US patients with PE are hospitalized.^4,5,6,7,8,9^ Anticoagulation remains the mainstay of treatment, including unfractionated heparin (UFH), low-molecular-weight heparins (LMWHs), and direct oral anticoagulants (DOACs).^10,11^ Of these treatments, UFH is the most labor intensive and carries the greatest risks despite being no more effective than the alternatives.^12^ In particular, its variable half-life, 2-phased elimination, and extensive binding to plasma proteins result in unpredictable pharmacokinetics that require nursing- or pharmacist-intensive protocols for laboratory monitoring.^13,14,15^ Despite monitoring, only 26% of patients with acute PE anticoagulated with UFH have been shown to be within the therapeutic range at 24 hours.^16^ Other studies have demonstrated an increased risk of bleeding with UFH.^17,18^ For these reasons, professional society guidelines recommend the use of LMWH or DOACs as the initial anticoagulation for patients with acute PE, unless a specific contraindication exists.^19,20^ Despite established guidelines, recent studies demonstrate increasing use of UFH in hospitalized patients with acute PE in the US.^21^ This qualitative study aimed to identify barriers and facilitators of guideline-concordant anticoagulation in patients hospitalized with acute PE.

## Methods

### Study Design

We used semistructured interviews among a purposive sample of emergency medicine and hospital medicine physicians in the US. The research team developed interview guides, piloted the guides among 3 emergency physicians, and iteratively revised the guides according to feedback (eAppendix in Supplement 1). Interviews began with questions about selection of anticoagulation in patients with acute PE being admitted to the hospital. The remainder of the interview guides were informed using 2 implementation science frameworks: the Consolidated Framework for Implementation Research (CFIR) and the Theoretical Domains Framework (TDF).^22,23,24^ The CFIR, which is composed of the domains outer setting, inner setting, innovation characteristics, individuals, and implementation process, offers constructs that help to systematically assess potential barriers and facilitators. The TDF focuses on individual-level behavioral change, a relatively small component of CFIR, but important within the context of clinician management decisions.^23^ We designed this study per current standards in qualitative methods and reported on all aspects of the Consolidated Criteria for Reporting Qualitative Research (COREQ) checklist (eTable 1 in Supplement 1).^25^ The Baystate Medical Center Institutional Review Board deemed the study as exempt from review in accordance with the Common Rule 45 CFR 45.104(d)(2i). Verbal consent was obtained from all interviewed physicians.

### Study Setting and Population

We used maximum variation sampling, a purposive sampling strategy that allows for a description of variability and identification of common or disparate themes based on purposefully chosen features.^26^ We selected this design on the basis of our hypothesis that preexisting patterns of anticoagulant use, whether at the regional, institutional, or individual level, are factors associated with anticoagulant choice and may affect clinician responses to barriers and facilitators of guideline-concordant anticoagulation. Thus, we recruited emergency medicine and hospital medicine physicians (hereafter, hospitalists) who met at least 2 of our sampling goals: to achieve balance in (1) UFH- and LMWH-predominant anticoagulation strategies and (2) hospital and physician characteristics. We aimed to recruit physicians across geographical regions, practice settings (academic, community, or hybrid [ie, both academic and community]), and length of time in practice. We included hospitalists because we hypothesized that inpatient hospital culture may play a role in anticoagulation choice.

We compiled a list of potential recruitment sites by examining the literature regarding site-specific dominant anticoagulation and using professional networks of PE researchers. Using this list, we recruited directly via email those participants who fulfilled the remaining sampling goals, inviting them to participate in a 30-minute interview to discuss the management of PE. After the preliminary analysis of interviews with emergency medicine physicians and hospitalists, we decided to triangulate the findings among a sample of interventional cardiologists and interventional radiologists (hereafter, interventionalists) who perform catheter-directed procedures for PE to cross-check the findings from our primary cohort. We used a truncated guide (eAppendix in Supplement 1) for these interviews. Interviews were conducted between February 1 and June 3, 2024.

### Data Collection

Using videoconferencing software (Zoom Video Communications), we conducted a single interview, approximately 30 minutes in duration, in a setting of the participant’s choosing. Two members of the research team were present for interviews, at least 1 of whom was a principal investigator (W.B.S. or L.M.W.). The audio was recorded. To mitigate social desirability bias, the interviewers communicated throughout the interview that there were no correct answers. Additionally, they explained that the aim of the study was to understand clinicians’ treatment of PEs and then obtained verbal consent.

Demographic data, including age, gender identity, race and ethnicity, number of years in practice, geographical location, practice setting, and presence of a PE response team (PERT) at the primary practice site, were collected. Race and ethnicity data were collected to demonstrate the diversity of the study population. Interviewers took field notes during the interviews to refine the interview guide and/or clarify nonverbal gestures. Interviewers conducted participant checking in real time to ensure they understood the participants’ points and perspectives. Transcripts were not returned to participants. Participants received a $25 Amazon gift card.

### Research Team 

There were 2 primary interviewers, a female emergency physician-researcher with extensive experience in qualitative research (L.M.W.) and a male emergency physician-researcher who underwent training in qualitative research and conducted proctored interviews with feedback before interviewing for the study (W.B.S.). Coinvestigators included a then-medical student with qualitative research experience (R.H.), 2 emergency medicine clinician-investigators (N.S., C.F.G.) who were trained in qualitative methods by a principal investigator, and a vascular cardiologist (G.D.B.). We held frequent meetings to discuss codes, themes, and biases.

### Data Analysis

Interviews were deidentified and transcribed verbatim. Qualitative data management software was used for coding and analysis (Dedoose version 8.1; SocioCultural Research Consultants LLC). We used reflexive thematic analysis, shifting between inductive and deductive modes.^27^ Five research team members read and re-read the transcripts.^27^ Each member reviewed the first transcript and created an initial coding framework using comparison and consensus. Each transcript was then coded independently by 2 members. We met and discussed codes and generated themes from the data, and transcripts were re-coded. The final codebook is available in eTable 2 in Supplement 1. Themes were reviewed and refined in the context of the full dataset and revised by discussion and consensus within the research team. After analysis, themes were mapped to CFIR and/or TDF for future implementation efforts. We obtained adequate sample size through our analysis of information power, in accordance with recommendations for reflexive thematic analysis.^27^

## Results

Of the 46 interviewees, 25 (54.3%) were emergency medicine physicians, 17 (37.0%) were hospitalists, and 4 (8.7%) were interventionalists who performed catheter-directed treatments for PE. Two individuals who were contacted to participate did not follow-up to schedule interviews. Participants included 13 women (28.3%) and 33 men (71.7%), with a median (IQR) age of 43 (36-50) years. Participant characteristics are provided in Table 1 and eTable 3 in Supplement 1. Interviews lasted a median (IQR) of 29 (25-32) minutes.

**Table 1.** Participant Characteristics

Characteristic	Participants, No. (%)		Total (n = 46)^a^
	Emergency physicians (n = 25)	Hospitalists (n = 17)	
Age, median (IQR), y	41 (33-47)	41 (36-52)	43 (36-50)
Gender identity			
Woman	5 (20.0)	9 (52.9)	13 (28.3)
Man	20 (80.0)	8 (47.1)	33 (71.7)
Race and ethnicity^b^			
Asian	8 (32.0)	7 (41.2)	18 (39.1)
Black	1 (4.0)	1 (5.9)	2 (4.3)
Hispanic	1 (4.0)	2 (11.8)	3 (6.5)
White	15 (60.0)	6 (35.3)	22 (47.8)
Prefer not to answer	0	1 (5.9)	1 (2.2)
Years in practice, median (IQR)	9 (7-19)	7 (5-21)	10 (6-20)
Geographical location			
Mid-Atlantic	4 (16.0)	2 (11.8)	6 (13.0)
Midwest	4 (16.0)	3 (17.6)	7 (15.2)
Northeast	2 (8.0)	6 (35.3)	9 (19.6)
South	8 (32.0)	3 (17.6)	13 (28.3)
West	7 (28.0)	3 (17.6)	11 (23.4)
Practice setting			
Academic	8 (32.0)	6 (35.3)	17 (37.0)
Community	9 (36.0)	7 (41.2)	17 (37.0)
Hybrid	8 (32.0)	4 (23.5)	12 (26.0)
Presence of PERT	14 (56.0)	6 (35.3)	23 (51.1)

Abbreviation: PERT, pulmonary embolism response team.

^a^ Including 4 interventionalists.

^b^ Self-described and not mutually exclusive.

Themes and subthemes are displayed in Table 2 and Table 3, and our theoretical framework is illustrated in the Figure. Many participants expressed a lack of strong opinion regarding the choice of anticoagulant for admitted patients with acute PE and relied on learned practices, citing therapeutic momentum (ie, continuing the treatment plan initiated by preceding clinicians) and/or institutional culture and support as factors associated with their practice behavior. Other central factors associated with anticoagulant choice were fear of decompensation or bleeding, misperceptions of pharmacology and adjunct procedures, and understanding of resource requirements.

**Table 2.** Illustrative Quotations of Central Themes in Initial Anticoagulation Choice

Themes and subthemes	Theoretical domain and construct (CFIR or TDF)	Representative quote (participant code)^a^
Agnostic to anticoagulation choice	Individuals: motivation (CFIR)/motivation (TDF)	“In my own practice, it almost never matters which anticoagulant I choose.” (E12)
Choices are equivalent to each other	Individuals: motivation (CFIR)/motivation (TDF)	“[The risk or benefit specific to LMWH or UFH] is really, sort of 6 of one and one-half dozen the other.” (H14)
Confidence in anticoagulation choice	Individuals (CFIR)/belief about capabilities: self-confidence, perceived competence (TDF)	“I’ve never felt like I needed guidance on like which anticoagulant to choose.” (E23)
Choice is “not that complicated”	Individuals (CFIR)/belief about capabilities: self-confidence, perceived competence (TDF)	“Choosing an anticoagulant is not a decision I lose sleep over. This one is pretty easy because all the options are generally pretty good.” (H10)
Defer choice to hospitalist or consultant	Individuals: opinion leaders (CFIR)/social or professional role and identity: professional boundaries, professional role (TDF)	“We use a lot of [UFH]….I don’t know if it’s the surgeons or the interventionalists….I don’t know who’s really fussy about the low-molecular-weight heparin, but we are heavy on UFH so it’s fine with me. I just need to keep the patients moving through the system and keep the upstairs doctors happy.” (E8)
Inertia of learned practice	Individuals (CFIR)/behavioral regulation: breaking habit (TDF)	“I guess it’s just kind of what I was taught in residency, and I’ve never really wavered from that kind of thing.” (E22)
		“You know old habits die hard, right?…we have used UFH for decades right prior to those newer medications. I think that a lot of times can just be because of familiarity with use and you’re more comfortable with it.” (E9)
Therapeutic momentum	Individuals: motivation (CFIR)/reinforcement (TDF)	“The emergency room provider has already started them on something. So, then we just have to continue that…if they’re on a heparin drip, then we continue that. If they’re on LMWH, then we continue that, and then the next day or so is when we start them on oral anticoagulation.” (H15)
Institutional culture and support	Inner setting: culture (CFIR)/social influences: group norms; modeling (TDF)	“If they’re going to be admitted, the preference is LMWH over UFH. That’s been slow to change, but there is some push coming from somewhere. I’m not exactly sure why, just thinking it’s a preferred agent in terms of possibly efficacy.” (H6)
Peer pressure	Inner setting: culture (CFIR)/emotion, beliefs about consequences, reinforcement: punishment (TDF)	“I once stepped on a nail because I tried to give somebody a shot of LMWH and admit them to the floor for their PE and got an angry email afterwards about that, ‘we don’t do that here.’’” (E12)
Anticipation of inpatient or consultant preference	Inner setting: relational connectedness (CFIR)	“It’s mostly driven by the culture of the institution that I work at…it’s what the inpatient team expects [giving UFH].” (E23)

Abbreviations: CFIR, Consolidated Framework for Implementation Research; ED, emergency department; LMWH, low-molecular-weight heparin; PE, pulmonary embolism; TDF, Theoretical Domains Framework; UFH, unfractionated heparin.

^a^ Participant specialty, location, and practice setting are provided in eTable 3 in Supplement 1.

**Table 3.** Illustrative Quotations of Anticoagulation-Specific Factors

Themes and subthemes	Theoretical domain and construct (CFIR/TDF)	Representative quote (participant identification)^a^
**Factors associated with UFH-dominant approach**		
Fear of decompensation	Individuals (CFIR)/knowledge; emotion: fear (TDF)	“If there is clinical decompensation and a decision to do some sort of an intervention, the [UFH] can be basically stopped or reversed and stopped.” (E22)
		“I think the reason that we do a heparin drip at my institution has a lot to do with this cultural idea that what if they need an intervention or what if they have a bleed? And so this is the fastest way to stop the anticoagulation.” (H16)
Misperception of pharmacology of anticoagulants	Individuals: capability (CFIR)/ knowledge (TDF)	“It’s an [intravenous] drip. So, in my mind, it’s gonna be quicker.” (E19)
Misperception of anticoagulation role in catheter-directed treatments	Individuals: capability (CFIR)/knowledge (TDF)	“[Using UFH] we have the option of stopping if they’re gonna get a thrombectomy or other intervention, and our larger facility is pushing more and more for thrombectomy approach to a lot of the intermediate and higher risk patients.” (E4)
**Factors associated with LMWH-dominant approach**		
Understanding of UFH as resource intensive	Innovation: relative; advantage; individuals: recipient centeredness(CFIR)/belief about consequences (TDF)	“It’s much more to manage for the hospital, right? The nurse has to manage drips, you have to take care of the PTT, the patient’s attached to a line that requires everyone running through it…there is a lot of nursing considerations as well.” (H11)

Abbreviations: CFIR, Consolidated Framework for Implementation Research; LMWH, low-molecular-weight heparin; PTT, partial thromboplastin time; TDF, Theoretical Domains Framework; UFH, unfractionated heparin.

^a^ Participant specialty, location, and practice setting are provided in eTable 3 in Supplement 1.

**Figure.**
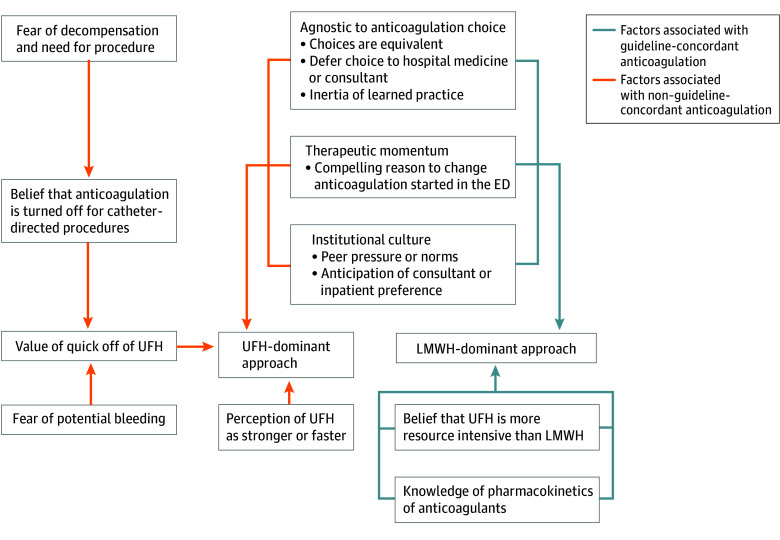
Central Theme Relationships ED indicates emergency department; LMWH, low-molecular-weight heparin; UFH, unfractionated heparin.

### Agnostic to Anticoagulation Choice

Although all participants indicated routine use of either LMWH or UFH, on probing the rationale underpinning their practice, many participants were found to be agnostic to the anticoagulant used and believed their choice of in-hospital anticoagulation did not matter. This perception appeared to be associated with the belief that all anticoagulants have similar effectiveness and risk profiles aside from the perceived “quick off” (ie, fast wearing off) of UFH. The routine use of one anticoagulant over another was largely attributed to either institutional culture or inertia of learned practice (ie, the inherited practice pattern generally learned in training) rather than to strong feelings about effectiveness or risk. Both emergency medicine physicians and hospitalists expressed deference to each other’s expertise or to consultants regarding initial anticoagulation. As a result, many participants reported choosing UFH to allow the physician who would ultimately treat the patient more flexibility in changing the anticoagulant.

### Therapeutic Momentum

Therapeutic momentum, defined as the reluctance to step down or withdraw therapy when further continuation is not supported by evidence, played a role in anticoagulation continuation across transitions of care.^28^ Emergency medicine physicians reported wanting to maintain the flexibility of changing the anticoagulant (with UFH) for hospitalists; however, hospitalists who preferred LMWH reported rarely switching patients started on UFH to LMWH or a DOAC until the patient was nearing discharge. This decision was largely attributed to convenience, timing of transitions, and the desire to reduce the number of anticoagulation transitions.

### Institutional Culture and Support

Participants uniformly discussed an unwritten institutional culture of using either an LMWH-dominant or a UFH-dominant approach to guide their practice. Anticipation of inpatient or consultant preference was the basis for anticoagulant selection for many emergency medicine physicians and hospitalists. Although aligning their practice patterns with institutional culture was important, no participants reported having formal guidelines for hospitalized patients. Participants reported PERT input existed only for the most severe PEs, leaving them uncertain about anticoagulation for non–high-risk PEs. Participants indicated that availability of a PERT and an institutional ability to perform catheter-directed treatments were reasons for their UFH use even in patients for whom a PERT was not indicated. However, when we interviewed members of inpatient teams and triangulated among interventionalists, we found they typically continued the previously initiated anticoagulant for the duration of the inpatient stay even if their overarching preference was for LMWH over UFH.

### Fear of Decompensation

Fear of decompensation and/or bleeding from the PE was a central factor associated with UFH use cited by participants. Participants voiced less concern for or knowledge of iatrogenic bleeding caused by the anticoagulant and instead worried about how to manage bleeding should it arise. Those who used an LMWH-dominant approach did not voice this fear, noting that few patients ultimately require catheter-directed treatment or rescue therapy. Furthermore, they reported working in systems in which LMWH was not a contraindication to either thrombolysis or catheter-directed treatment. Nearly all participants specifically cited the quick off of UFH, valuing the short half-life of UFH, and cited that UFH afforded the ability for any procedures a patient may need even if none was anticipated.

### Misperception of Pharmacology of Anticoagulants

In many cases, participants reported choosing UFH because they perceived it to be stronger and have a quicker onset of action than other anticoagulants. The maxim “quick on, quick off” (ie, near-immediate achievement of therapeutic effect and fast wearing off once medication is stopped) was used nearly ubiquitously by participants. The perception among participants was that because UFH was given intravenously, it achieved therapeutic anticoagulation nearly immediately. The perception of UFH as stronger than other anticoagulants was particularly valued when clinicians were worried about substantial clot burden or potential decompensation. As an emergency medicine physician from a community practice in the South said, “[UFH] is an [intravenous] drip. So, in my mind, it’s gonna be quicker.”

Toward the end of many interviews, several participants reconsidered their statements on the quick on of UFH. For example, a hospitalist from a hybrid practice in the Midwest, who stated at the beginning of the interview, “You’re already anticoagulated as soon as you start [UFH] and there’s minimal follow up” later in the interview stated, “I would actually consider people who are on LMWH therapeutic more quickly than those who are on UFH, because you have to check…you know, because of needing to keep an eye on the PTTs [partial thromboplastin times]…sometimes people don’t really budge after their initial dosing.”

### Misperception of Anticoagulation Role in Catheter-Directed Treatments

Nearly all emergency medicine physicians and hospitalists believed anticoagulation was withheld, turned off, or reversed for catheter-directed treatments. The potential for a patient to receive a catheter-directed treatment was a powerful motivator to select UFH as an initial anticoagulant despite a lack of evidence that other anticoagulants are contraindicated or viewed as an impediment to catheter-directed intervention. To explore this finding, we engaged 4 interventionalists from locations that we previously sampled. All interventionalists reported that anticoagulation with LMWH was not a contraindication to catheter-directed treatments for PE. The 2 interventional cardiologists reported defaulting to UFH based on use in left heart catheterizations but were not opposed to LMWH.

### Understanding of UFH as Resource Intensive

Although many participants reported that UFH was the easy choice for the ordering physician, participants who used an LMWH-dominant approach emphasized UFH as resource intense for both nursing and patients. Many participants who primarily used a UFH-dominant approach later in the interview reconsidered the burden of laboratory draws and titration on patients and nursing. As a hospitalist from a hybrid practice in the Northeast said, “Every 6-hour PTTs [partial thromboplastin times]–patients hate it, and we hate it, too, because you have to get blood every 6 hours, and to monitor that PTT. And most of the time it takes a while to get therapeutic.”

## Discussion

In this qualitative study, we found that both emergency medicine physicians and hospitalists were largely agnostic to anticoagulation choice in the treatment of acute PE, and their preferences were principally based on inertia of learned practice, therapeutic momentum, and institutional culture and support. Fear and misperceptions of pharmacological properties and adjunct procedures were associated with a UFH-dominant approach, whereas understanding of the resource-intense nature of UFH was associated with an LMWH-dominant approach. These findings provide insight into the increase in UFH use among hospitalized patients with acute PE.

Deimplementation of low-value care involves both learning a new practice pattern and unlearning historical clinical practice.^29,30^ During interviews, both emergency medicine physicians and hospitalists routinely referred to inertia carried over from training (generally when discussing a habit of using UFH in patients admitted with PE). This inertia was, at least in part, due to a general indifference toward anticoagulation choice. Adoption of new practice patterns requires awareness of clinical guidelines and data. We noted 3 factors that may serve as barriers to seeking new information: participants’ (1) confidence in their anticoagulation choices, (2) belief that treatment of hospitalized patients was not complicated, and (3) perception that other clinicians had more expertise in this decision-making. As a result, physicians appeared to fall back on inherited practice and were unmotivated to seek new information regarding the anticoagulation of hospitalized patients with PE.

Many participants believed that UFH allowed flexibility for the downstream care team and that another specialty had expertise in anticoagulant selection. Additionally, hospitalists revealed that this practice may play a role in the overuse of UFH through therapeutic momentum. Despite the indifference and deference verbalized by many emergency medicine physicians, their initial selection of anticoagulant is the primary factor indicating the approach used throughout the remainder of the hospitalization. As a result, strategies targeting inpatients and hospitalists may be insufficient, and effective efforts to curb UFH overuse in hospitalized patients with acute PE must include emergency medicine physicians.

Institutional support for alternatives to UFH, particularly from consultants and interventionalists, was important to participants; however, few had clear knowledge of consultant preferences. Although some participants reported having protocols for outpatient treatment of PE and PERTs, all lacked formal guidance for most patients hospitalized with PE. Multidisciplinary engagement is critical in assuaging uncertainty and fear and overturning indifference in anticoagulation choice. The presence of a PERT alone is likely insufficient because data are mixed on the implications of a PERT for anticoagulation choice. Although some single-center retrospective analyses found increasing use of LMWH in patients after implementation of a PERT, other studies found the opposite.^31,32^ Multidisciplinary agreement on treatment pathways across the spectrum of PE and spanning the spectrum of care (ED visit to discharge) may help clinical decision-making.

Although most participants rationalized their anticoagulation choices by citing commonly held beliefs, several of these were misperceptions. Perhaps more interesting is that nearly all participants eventually identified their own cognitive dissonance during the interviews. For example, most participants used the phrase *quick on, quick off* regarding UFH and only later in the interviews reconsidered the time to therapeutic anticoagulation. Similarly, participants struggled with UFH bleeding risk, verbalizing an equivalent risk between UFH and LMWH and prioritizing reversibility over iatrogenic bleeding risk while also noting that patients treated with UFH seemed to have more frequent issues with bleeding. Additionally, UFH was considered to be easy for the ordering clinician; however, later in the interviews, participants often discussed the burden of UFH monitoring for other members of the care team, citing dosing adjustments and multiple additional blood draws for the patient. Although clinicians may possess knowledge of pharmacology and risks and benefits, they do not necessarily access this knowledge readily when making decisions and may rely on more pragmatic, fast-thinking heuristics when in the busy ED setting. These findings suggest that education alone is likely insufficient for changing initial anticoagulation choice. Implementation strategies integrating behavioral economics–based nudges into the workflow may aid task-saturated physicians in making more evidence-based decisions for patients with acute PE.

### Limitations

There are 3 notable limitations to this study. First, qualitative work is not intended to be generalizable to all practice settings or to summarize all potential perspectives. For example, we limited the sample to US-based physicians. However, we used a sampling strategy that produced a sample of physicians spanning the spectrum of practice patterns and settings in the US. This strategy allowed us to identify common themes despite variations in physician characteristics and practice environments. Second, although we undertook efforts to minimize social desirability bias, participants may have provided answers they felt interviewers would view favorably. Third, interpretation of transcripts using reflexive thematic analysis has intrinsic subjectivity in discerning meaning from data. Multiple members of the research team reviewed each transcript, and we met regularly to examine our subjectivity and context and how these may have affected our interpretation of the data.

## Conclusions

This qualitative study identified common factors associated with anticoagulation choice in acute PE, spanning physician practice settings and specialty. Common barriers and facilitators to use of guideline-concordant anticoagulation included agnosticism regarding choice of anticoagulant, the inertia of learned practice, and therapeutic momentum after anticoagulation initiation. Institutional culture and support were associated with use of the dominant anticoagulation strategy. Efforts to improve the quality and value of treatment of patients with PE should target these barriers and facilitators to guideline-concordant anticoagulation.

## Notes

Supplement 1. **eAppendix.** Interview Guides **eTable 1.** Steps Taken to Ensure Methodological Rigor **eTable 2.** Codebook **eTable 3.** Participant Details

Supplement 2. Data Sharing Statement
