# Supplementary material for: Factors in Initial Anticoagulation Choice in Hospitalized Patients With Pulmonary Embolism
Source: JAMA Netw Open. 2025 Jan 3;8(1):e2452877. doi: 10.1001/jamanetworkopen.2024.52877 (PMC11699532; doi:10.1001/jamanetworkopen.2024.52877)
Supplement: Supplement 1. — eAppendix. Interview Guides eTable 1. Steps Taken to Ensure Methodological Rigor eTable 2. Codebook eTable 3. Participant Details [file jamanetwopen-e2452877-s001.pdf]

Supplemental Online Content

Stubblefield WB, Helderman R, Strokes N, et al. Factors in initial anticoagulation choice in hospitalized patients with pulmonary embolism. *JAMA Netw Open*. 2025;8(1):e2452877. doi:10.1001/jamanetworkopen.2024.52877

eAppendix. Interview Guides

eTable 1. Steps Taken to Ensure Methodological Rigor

eTable 2. Codebook

eTable 3. Participant Details

This supplemental material has been provided by the authors to give readers additional information about their work.

© 2025 Stubblefield WB et al. *JAMA Network Open*

eAppendix. Interview Guides

Interview guide for emergency physicians

Thank you so much for taking the time to meet with us. My name is _______ and I'm an emergency physician and researcher. [XXX] is also joining us today as part of the research team. Today we will be talking about the treatment of pulmonary embolism, specifically anticoagulation. I will be asking you questions about your perspective and thoughts - there are no right or wrong answers. We will protect the confidentiality of your research information by keeping any demographic information in an IRB-approved online database that only research staff will have access to. Conversations from the interview will be recorded but any identifying or personal information will be removed from the conversation before any data analysis happens so nobody other than those present right now will be able to identify what you say. By agreeing to continue with this interview you are consenting to participate in this research study with the understanding that you are free to withdraw at any time. If at any time you wish to discontinue your participation, you may leave the interview or ask us to stop contacting you and it will not result in any penalty or loss of benefits to which you are otherwise entitled. You will receive a $25 Amazon gift card as a sign of our appreciation. Do you have any questions before we get started?

Do I have your permission to begin and record the interview?

We are doing these interviews to learn about treatment decisions for patients diagnosed with acute pulmonary embolism in the Emergency Department, particularly anticoagulation choice. I want you to think about the last patient you diagnosed with an acute PE in the Emergency Department who you admitted to the hospital. Thinking about that patient, how did you go about treating their PE?

Probes: Was this usual practice for you or are there cases in which you would have chosen a different anticoagulant? (e.g. in hemodynamically stable patients). Tell me more about those cases?

How do you decide which anticoagulant to initiate from the ED?

Probes: How did you develop this practice pattern? Is there an institutional protocol? (if yes, tell me more about it - how do you access it, how did you come to learn about it etc). When do you use LMWH versus UFH?

How confident are you that you will be able to successfully choose the appropriate anticoagulant for each patient?

How complicated is determining the appropriate initial anticoagulant for each patient? (innovation: complexity)

Probes: What about in patients with renal dysfunction or extremes of BMI/body weight?

Are there things that could make anticoagulant choice simpler? (If needed - can ask about e.g. protocol, institutional guidance)

What supports (e.g. online resources, toolkit, decision support) are available to help you choose an anticoagulant? (innovation: design)

Probes: Tell me more about them. Do you have any clinical decision support to help you choose an anticoagulant i this case? (how do you feel about it?)

- ○ If no, what do you think you would find helpful? How would you access it?

Are there any cases in which you would start a direct oral anticoagulant in patients you are admitting to the hospital?

When considering anticoagulation in hemodynamically stable patients with evidence of right heart strain on imaging **AND** elevated cardiac biomarkers, which anticoagulant do you use?

- What do you see as the benefits to using [primary anticoagulant they use]
  - *Probe - [if discussed 'quick on'] What does 'quick on, quick off' mean to you?"*
  - *Probe - [If discussed 'quick on'] How important is fast onset of action to therapeutic anticoagulation? (if they don't give a time frame, how long would be too long? 1 hour, 8 hours?)*
    - *If you were to discover that the average time until therapeutic heparin is 8 hours?* [knowledge gap - UFH time delay]
- What do you see as the potential harms to using [primary anticoagulant they use]
  - *Probe - What about bleeding outcomes - do you worry more about UFH or LMWH?*

© 2025 Stubblefield WB et al. *JAMA Network Open*

- Do you perceive any harms to LMWH in this subgroup?

© 2025 Stubblefield WB et al. *JAMA Network Open*

    - *Probe - Risk of LMWH in thrombolysis*
- How do you balance these benefits and harms in this subgroup?
- What would make you more confident in using LMWH in this subgroup?

Interview Guide – Hospital Medicine

Thank you so much for taking the time to meet with us. My name is _______ and I'm an emergency physician and researcher. [XXX] is also joining us today as part of the research team. Today we will be talking about the treatment of pulmonary embolism, specifically anticoagulation. I will be asking you questions about your perspective and thoughts - there are no right or wrong answers. We will protect the confidentiality of your research information by keeping any demographic information in an IRB-approved online database that only research staff will have access to. Conversations from the interview will be recorded but any identifying or personal information will be removed from the conversation before any data analysis happens so nobody other than those present right now will be able to identify what you say. By agreeing to continue with this interview you are consenting to participate in this research study with the understanding that you are free to withdraw at any time. If at any time you wish to discontinue your participation, you may leave the interview or ask us to stop contacting you and it will not result in any penalty or loss of benefits to which you are otherwise entitled. You will receive a $25 Amazon gift card as a sign of our appreciation. Do you have any questions before we get started?

Do I have your permission to record the interview?

We are doing these interviews to learn about treatment decisions for patients diagnosed with acute pulmonary embolism, particularly anticoagulation choice. I want you to think about the last patient you treated who was admitted for their PE. Thinking about that patient, how did you go about treating their PE?

- *Probe: Was this usual practice for you or are there cases in which you would have chosen a different anticoagulant? (e.g. in hemodynamically stable patients)*
- *Tell me more about those cases?*

How do you decide which anticoagulant to use?

Probes: How did you develop this practice pattern? Is there an institutional protocol? (if yes, tell me more about it - how do you access it, how did you come to learn about it etc). When do you use LMWH versus UFH?

Can you tell me about any times you change the initial anticoagulant started by the ED team?

How confident are you that you will be able to successfully choose the appropriate anticoagulant for each patient?

How complicated is determining the appropriate initial anticoagulant for each patient? (innovation: complexity)

Probes: What about in patients with renal dysfunction or extremes of BMI/body weight?

Are there things that could make anticoagulant choice simpler? (If needed - can ask about e.g. protocol, institutional guidance)

What supports (e.g. online resources, toolkit, decision support) are available to help you choose an anticoagulant? (innovation:: design)

Probes: Tell me more about them. Do you have any clinical decision support to help you choose an anticoagulant i this case? (how do you feel about it?)

- If no, what do you think you would find helpful? How would you access it?

Are there any cases in which you would start a direct oral anticoagulant in patients you are admitting to the hospital?

© 2025 Stubblefield WB et al. *JAMA Network Open*

When considering anticoagulation in hemodynamically stable patients with evidence of right heart strain on imaging **AND** elevated cardiac biomarkers, which anticoagulant do you use?

- What do you see as the benefits to using [primary anticoagulant they use]
  - *Probe - [if discussed 'quick on'] What does 'quick on, quick off' mean to you?"*
  - *Probe - [If discussed 'quick on'] How important is fast onset of action to therapeutic anticoagulation? (if they don't give a time frame, how long would be too long? 1 hour, 8 hours?)*
    - *If you were to discover that the average time until therapeutic heparin is 8 hours?* [knowledge gap - UFH time delay]
- What do you see as the potential harms to using [primary anticoagulant they use]
  - *Probe - What about bleeding outcomes - do you worry more about UFH or LMWH?*
- Do you perceive any harms to LMWH in this subgroup?
  - *Probe - Risk of LMWH in thrombolysis*
- How do you balance these benefits and harms in this subgroup?
- What would make you more confident in using LMWH in this subgroup?

Interview Guide – Interventional Radiology/Cardiology

Thank you so much for taking the time to meet with us. My name is _______ and I'm an emergency physician and researcher. [XXX] is also joining us today as part of the research team. Today we will be talking about the treatment of pulmonary embolism, specifically anticoagulation. I will be asking you questions about your perspective and thoughts - there are no right or wrong answers. We will protect the confidentiality of your research information by keeping any demographic information in an IRB-approved online database that only research staff will have access to. Conversations from the interview will be recorded but any identifying or personal information will be removed from the conversation before any data analysis happens so nobody other than those present right now will be able to identify what you say. By agreeing to continue with this interview you are consenting to participate in this research study with the understanding that you are free to withdraw at any time. If at any time you wish to discontinue your participation, you may leave the interview or ask us to stop contacting you and it will not result in any penalty or loss of benefits to which you are otherwise entitled. You will receive a $25 Amazon gift card as a sign of our appreciation. Do you have any questions before we get started?

We are doing these interviews to learn about anticoagulation treatment decisions for patients with acute pulmonary embolism, particularly anticoagulation choice.

Can you tell me a little bit about the role of anticoagulation in patients undergoing catheter-directed treatment for PE?

What aspects of anticoagulation are important to you?

- *Probe if needed: reversibility, therapeutic anticoagulation, monitoring*

Does your hospital system/institution have a protocol for anticoagulation of patients who have acute PE?

- *Probe if needed: Is there a protocol that directs what to do if it is possible may go for intervention?*

Do you weigh in on anticoagulation in patients with acute PE?

- Probes: How common is it you weigh in on anticoagulation? What anticoagulant do you recommend? What do you see as advantages or disadvantages?

If not mentioned above, how do you feel about doing procedures on patients who previously received a therapeutic dose of LMWH?

- *What do you see as advantages? What about disadvantages?*

© 2025 Stubblefield WB et al. *JAMA Network Open*

If not mentioned above, How do you feel about doing procedures on patients who previously received a therapeutic dose of DOAC?

- *What do you see as advantages? What about disadvantages?*

eTable 1. Steps Taken to Ensure Methodological Rigor

	Purpose	Strategies applied in our study to achieve rigor
**Credibility**	To establish confidence in the results	Interview protocol (guide and process) tested in pilot interviews
		Trained primary interviewer and investigators – PI LMW is an experienced qualitative researcher and methodologist. She trained PI WBS with didactic sessions, case studies, and hands-on training. WBS conducted pilot proctored interviews with feedback from LMW and the interviewee. Co-investigator RH had previously been trained in qualitative methods by LMW for another study. LMW trained co-investigators CFG and NS via didactics, proctored data-analysis, and review. GDB and DRV have prior involvement in qualitative studies and served as content experts (pulmonary embolism and implementation science)
		Participant checking in real time – Allows participants to correct errors and challenge what are perceived as wrong interpretations
**Dependability**	To ensure the findings are repeatable within the same cohort of participants, coders, and context	Rich description of the study methods: We created a detailed draft of the study protocol. The primary interviewer did not have a working relationship with the interviewee, to minimize social desirability bias
**Confirmability**	To extend confidence that the results were derived from the data and would be confirmed by other researchers	Reflexivity: We regularly met to discuss our biases and assumptions. We included a non-emergency physician as part of the research team to mitigate emergency medicine-centric biases. Additionally, we included 2 members of the research team who do not have content-area expertise in venous thromboembolism to ensure diversity of perspectives.
		Triangulation: We triangulated sources, including with prior literature, among hospital medicine physicians and interventional cardiologists and interventional radiologists
		Information power: The use of theoretical saturation in reflexive thematic analysis is problematic. As a result, we assessed information power
		Thick description: we used probes in the semi-structured interviews to enhance the description

eTable 2. Codebook

**Fear driving UFH use**
"Just in case" / "what if..."
Fear of decompensation
No DOAC as inpatient "just in case"
Fear of needing to stop anticoagulation
Difficulty "turning-off" LMWH
Difficulty reversing LMWH
**Agnostic to anticoagulation choice**
All the options are fine/similar
Anticoagulants similar with regard to bleeding risk
"It doesn't matter"
Never compared anticoagulant risks/benefits
Agnostic to time to therapeutic anticoagulation
Agnostic to UFH risks
**Defer treatment to “expert” and/or downstream clinician**
Try to anticipate inpatient choice
Defer to PERT team/interventional
Defer to consultants, in right heart strain
Feel PERT activation influences choice
Perception that PERT team/IR wants UFH
DOAC only after inpatient consult
Wants hematology consult
Trusts consultants
Consultant blessing of LMWH would help
**UFH in anticipation of what another physician/service wants**
Belief that consultants want to "turn off" UFH for procedure
UFH in case CDT procedure needed
Belief that catheter therapies require UFH
Belief you have to stop anticoagulation for intervention
**Peer pressure**
Institutional/consultant pushback for UFH (use LMWH)
Pharmacy pushback to use LMWH
**UFH resource intensive**
Hassle of UFH or nursing
LMWH less complicated
Perceive hospitalists as focusing on ease of dosing
LMWH lower resource utilization
UFH requires frequent labs (patient-centered)
**Value "quick off" of UFH**
"Just in case"

© 2025 Stubblefield WB et al. *JAMA Network Open*

DOACs not easily reversible
For patients needing surgical procedures
For patients who are high risk and anticoagulated before CTPA
Quick off leaves options open for inpatient teams
LMWH commits to 12-24 hour anticoagulation
Start UFH and inpatient team can transition
Risk of bleed less important than reversibility
Worried about handling bleeding on LMWH
Risk of bleed, use UFH
**Perceive UFH "quick on, quick off"**
Believe UFH therapeutic in a couple of hours
Particularly for procedures/lytics
Clot burden drives anticoag decision
Particularly important in big PEs
Say quick on, quick off and realize only quick off
Want anticoagulation quickly, particularly in sick patients
**Anticoagulation choice is "not that complicated"**
Doesn't perceive need for guidance (for self)
Only 3 options
**Anticoagulation choice is multifactorial/unorganized**
CDS would be helpful
CDS would make choice more objective
Could signal institutional priority/permission
Order set reminds of renal function
CDS would decrease disagreement with other services
**Inertia of learned practice "How I've always done it"**
Clinical experience
Inherited practice
Inertia from residency drives anticoagulant choice
Trained to use LMWH first
Used UFH in residency/training
**Knowledge**
Dissonance between perception of UFH titration and risk of overdose/bleed
Knowledge about LMWH and equivalent outcomes
Knowledge gap of pharmacokinetics of "quick on" UFH
Lack of knowledge about LMWH
Lack of knowledge of time of onset
**Fear of UFH**
Fear of UFH causing bleed
Has seen bad bleeding outcomes with UFH
Patients on UFH in "high-risk" state and "pre-disposed" to bleeding

© 2025 Stubblefield WB et al. *JAMA Network Open*

**Institutional culture/support**
Practice pattern driven by keeping relationship with inpatient/institution status quo
Institutional culture is LMWH usually
Know hospitalists prefer LMWH
Institutional culture of UFH over LMWH
Pressure from hospitalist to use UFH
Got reprimanded for using LMWH
Institutional policies can help change practice (generally)

Abbreviations: Direct oral anticoagulation (DOAC); Low Molecular Weight Heparin (LMWH); Unfractionated Heparin (UFH); Pulmonary Embolism Response Team (PERT); Interventional Radiology (IR); Catheter directed therapy (CDT); Computed Tomography Pulmonary Angiogram (CTPA); Clinical decision support (CDS); Pulmonary Embolism (PE)

© 2025 Stubblefield WB et al. *JAMA Network Open*

eTable 3. Participant Details

Participant identifier	Brief description
E1	Emergency physician; West; Hybrid practice/Critical Access
E2	Emergency physician; West; Hybrid practice
E3	Emergency physician; West; Hybrid practice
E4	Emergency physician; South; Community practice/Critical Access
E5	Emergency physician; West; Community practice
E6	Emergency physician; West; Hybrid practice
E7	Emergency physician; South; Community practice
E8	Emergency physician; Midwest; Academic practice
E9	Emergency physician; South; Academic
E10	Emergency physician; South; Academic
E11	Emergency physician; Midwest; Community
E12	Emergency physician; Midwest; Hybrid
E13	Emergency physician; South; Community
E14	Emergency physician; Mid-Atlantic; Hybrid
E15	Emergency physician; West; Academic
E16	Emergency physician; Northeast; Hybrid
E17	Emergency physician; South; Academic
E18	Emergency physician; West; Academic
E19	Emergency physician; South; Community
E20	Emergency physician; Mid-Atlantic; Hybrid
E21	Emergency physician; South; Community
E22	Emergency physician; Mid-Atlantic; Hybrid
E23	Emergency physician; Mid-Atlantic; Hybrid
E24	Emergency physician; Northeast; Academic
E25	Emergency physician; Midwest; Academic
H1	Hospitalist; West; Community

© 2025 Stubblefield WB et al. *JAMA Network Open*

H2	Hospitalist; West; Hybrid
H2	Hospitalist; Northeast; Community
H3	Hospitalist; South; Community
H4	Hospitalist; Northeast; Community
H5	Hospitalist; Northeast; Academic
H6	Hospitalist; Midwest; Academic
H7	Hospitalist; South; Academic
H8	Hospitalist; Northeast; Hybrid
H9	Hospitalist; West; Community
H10	Hospitalist; Midwest; Hybrid
H11	Hospitalist; Mid-Atlantic; Community
H12	Hospitalist; Northeast; Academic
H13	Hospitalist; Midwest; Hybrid
H14	Hospitalist; Northeast; Community
H15	Hospitalist; Northeast; Academic
H16	Hospitalist; Midwest; Academic
H17	Hospitalist; South; Community
I1	Interventionalist; South; Academic
I2	Interventionalist; West; Community
I3	Interventionalist; South; Academic
I4	Interventionalist; Northeast; Academic

© 2025 Stubblefield WB et al. *JAMA Network Open*

© 2025 Stubblefield WB et al. *JAMA Network Open*
