# Supplementary material for: Factors in Initial Anticoagulation Choice in Hospitalized Patients With Pulmonary Embolism
Source: JAMA Netw Open. 2025 Jan 3;8(1):e2452877. doi: 10.1001/jamanetworkopen.2024.52877 (PMC11699532; doi:10.1001/jamanetworkopen.2024.52877)
Supplement: Supplement 2. — Data Sharing Statement [file jamanetwopen-e2452877-s002.pdf]

Data Sharing Statement

Stubblefield. Factors in Initial Anticoagulation Choice in Hospitalized Patients With Pulmonary Embolism. *JAMA Netw Open*. Published January 03, 2025.
doi:10.1001/jamanetworkopen.2024.52877

Data
Data available: Yes
Data types: Other (please specify)
Additional Information: De-identified data
How to access data: The data that support the findings of this study are available from the corresponding author, [LMW], upon reasonable request.
When available: With publication

Supporting Documents
Document types: None

Additional Information
Who can access the data: Researchers whose proposed use of the data has been approved
Types of analyses: any purpose
Mechanisms of data availability: with investigator support
